# Performance of Glutamate Dehydrogenase and Triose Phosphate Isomerase Genes in the Analysis of Genotypic Variability of Isolates of *Giardia duodenalis* from Livestocks

**DOI:** 10.1155/2013/875048

**Published:** 2013-11-06

**Authors:** Natália M. N. Fava, Rodrigo M. Soares, Luana A. M. Scalia, Evanguedes Kalapothakis, Isabella F. Pena, Carlos U. Vieira, Elaine S. M. Faria, Maria J. Cunha, Talles R. Couto, Márcia Cristina Cury

**Affiliations:** ^1^Laboratório de Parasitologia, Instituto de Ciências Biomédicas, Universidade Federal de Uberlândia, 1720 Avenida Pará, Umuarama Campus, 38400-902 Uberlândia, MG, Brazil; ^2^Departamento de Veterinária Preventiva e Saúde Animal, Faculdade de Medicina Veterinária e Zootecnia, Universidade de São Paulo, Avenida Prof. Dr. Orlando Marques de Paiva, 87-Cidade Universitária, 05508-270 São Paulo, SP, Brazil; ^3^Laboratório de Biotecnologia e Marcadores Moleculares, Departamento de Biologia Geral, Instituto de Ciências Biológicas, Universidade Federal de MG, 6627 Avenida Antônio Carlos, Pampulha Campus, 31270-901, Belo Horizonte, MG, Brazil; ^4^Médico Veterinário—Instituto de Ciências Biomédicas, 1720 Avenida Pará, Umuarama Campus, 38400-902 Uberlândia, MG, Brazil

## Abstract

*Giardia duodenalis* is a small intestinal protozoan parasite of several terrestrial vertebrates. This work aims to assess the genotypic variability of *Giardia duodenalis* isolates from cattle, sheep and pigs in the Southeast of Brazil, by comparing the standard characterization between glutamate dehydrogenase (*gdh*) and triose phosphate isomerase (*tpi*) primers. Fecal samples from the three groups of animals were analyzed using the zinc sulphate centrifugal flotation technique. Out of 59 positive samples, 30 were from cattle, 26 from sheep and 3 from pigs. Cyst pellets were stored and submitted to PCR and nested-PCR reactions with *gdh* and *tpi* primers. Fragment amplification of *gdh* and *tpi* genes was observed in 25 (42.4%) and 36 (61.0%) samples, respectively. Regarding the sequencing, 24 sequences were obtained with *gdh* and 20 with *tpi*. For both genes, there was a prevalence of E specific species assemblage, although some isolates have been identified as A and B, by the *tpi* sequencing. This has also shown a larger number of heterogeneous sequences, which have been attribute to mixed infections between assemblages B and E. The largest variability of inter-assemblage associated to the frequency of heterogeneity provided by *tpi* sequencing reinforces the polymorphic nature of this gene and makes it an excellent target for studies on molecular epidemiology.

## 1. Introduction

*Giardia duodenalis* (*Giardia lamblia*, *Giardia intestinalis*) is a small intestinal parasitic protozoan of several terrestrial vertebrates and has been described throughout the world in livestock, regardless of their capability, sex, and age. Prevalence rates, in humans and in animals, may vary among countries, probably due to differences in the management, weather, and type of study conducted to diagnose the infections [1].

The protozoan is currently considered as a complex of species, due to the existing genetic differences among isolates infecting different hosts [2]. Eight different assemblages have been attributed to this complex but with similar morphologies. Isolates recovered from human feces and feces from other mammal species belong to two genetic groups, called Polish and Belgian in Europe and assemblages A and B in Australia [3, 4]. Six other assemblages have been considered as species-specific (C to H) [5], with C and D prevailing in dogs, E in ungulates, F in cats, G in rats, and assemblage H in marine vertebrates [6]. 

Substantial differences found in glutamate dehydrogenase (*gdh*), *β*-giardin, triose phosphate isomerase (*tpi*), and SSUr-RNA genes were crucial for the recognition of intraspecific variations, which were called assemblages. These genes have a discriminatory ability, which is enough to identify intra-assemblage variations, used for genotyping, but, mainly, for subgenotyping of isolates [7, 8].

Cattle, pigs, and sheep are susceptible to infections with species-specific assemblage E and also with zoonotic infections of *Giardia duodenalis* assemblages A and B [9–13].

For some time, most studies on genotypic characterization were only conducted with a single gene locus; however, studies have shown inconsistent results when distinct loci are sequenced. Thus, the genotyping of more than one gene may improve the attribution of the assemblage to its respective isolate, helping in the understanding of the epidemiology of giardiasis [8]. 

This work has the purpose of assessing the genotypic variability among isolates of *Giardia duodenalis *from cattle, pigs, and sheep from the southeastern region of Brazil, when comparing the pattern of characterization between primers targeting glutamate dehydrogenase (*gdh*) and triose phosphate isomerase (*tpi*) genes.

## 2. Materials and Methods

### 2.1. Population of Study

This work has been approved by “Comitê de Ética na Utilização de Animais da Universidade Federal de Uberlândia” (CEUA-UFU) (Ethics Committee on Animal Use of the Federal University of Uberlândia), protocol 003/12.

Cattle, pigs, and sheep, with an age range of 0 to 10 months, both males and females, and with different breeds from the microregion of Uberlândia, Minas Gerais state, southeastern region of Brazil have been included in the study.

Cattle were distributed in 17 farms; 5 of them were Holstein cattle (PO) (for milk production) and 12 with Girolando cattle, were milk and beef production. Sheep came from a single farm with Corriedale breed with meat and wool production, and pigs came from 10 commercial farms, with Landrace breed.

### 2.2. Fecal Samples

#### 2.2.1. Sample Collection

Feces were collected individually straight from the rectal ampulla of cattle and sheep. Regarding pigs, samples were collected in pools, straight from the floor of the stalls, as they were grouped in lots of 30 to 40 animals, according to their age. Each pool was considered as a sample. 

Due to the intermittent pattern of elimination of *Giardia duodenalis* cysts, three fecal samples from each cow and sheep were collected every other day in order to increase the reliability of the study. In pigs, samples were collected once due to the sanitary management of the farm. 

Feces were collected and stored in plastic bags identified with the number of each animal or lot, the name of the farm, and the date of collection and were sent to Laboratório de Parasitologia (laboratory of parasitology) of Universidade Federal de Uberlândia (UFU) for processing.

#### 2.2.2. Sample Processing

Samples were considered positive for *Giardia duodenalis *cysts using the zinc sulphate centrifugal flotation technique [14]. Slides and coverslips with positive samples were washed with sodium phosphate buffered saline (PBS), pH 7.2, and then transferred to polystyrene microtubes. These were submitted to three centrifugations at 10,000 xg for ten minutes each. In each centrifugation, the supernatant was discarded, and a new PBS was added. Cyst pellets were stored at −20°C for later use.

### 2.3. DNA Extraction

After resuspension in 500 *μ*L of lysis buffer (10 mM Tris-HCl, pH 8.0; 25 mM EDTA, pH 8.0; 100 mM NaCl; 1% SDS), cyst pellets were submitted to three cycles of freezing/thawing. Ten mg/mL of proteinase K was added to the liquid product, and then it was incubated at 37°C for 12 hours. DNA was extracted following the phenol-chloroform protocol for DNA extraction described by Sambrook et al., 1989 [15]. Negative controls were used in each extraction group. 

### 2.4. Polymerase Chain Reaction (PCR)

In order to amplify the fragments of glutamate dehydrogenase (*gdh*) and triose phosphate isomerase (*tpi*) genes, primers developed by Cacciò et al. [8] and Sulaiman et al. [7] were used. 

The fragment of 530 base pairs of *gdh *gene was obtained using both external (Gdh 1 and Gdh 2) and internal primers (Gdh 3 and Gdh 4). 

In order to amplify the fragment of *tpi *gene (530 pb), external primers (AL3543 and AL3546) and internal primers (AL3544 and AL3545) were used.

PCR and nested-PCR reactions were performed in Mastercycler pro thermocycler (Eppendorf, Brazil) according to the protocols described by Cacciò et al. [8] and Sulaiman et al. [7]. 

Bands of interest were visualized through the agarose gel electrophoresis technique at 2% (P/V), and stained with ethidium bromide at 0.5 *μ*g/mL with further observation using an ultraviolet transilluminator. Aliquots of 8 *μ*L of the amplified sample were analyzed.

### 2.5. Sequencing and Alignment of DNA

Positive nested-PCR products were purified with Sephacryl 400 resin (Ilustra-MicroSpin S400 HR Columns) and sequenced in a single direction. Reactions were performed in a Mastercycler pro thermocycler (Eppendorf, Brazil) using Big Dye terminator V.3.1 Cycle Sequencing Kit (Applied Biosystems, Foster City, CA, USA). Products were read using the automatic sequencer ABI 3130 Genetic Analyzer (Applied Biosystems, Foster City, CA, USA).

The quality of the partial sequences and the clustering of the fragments were obtained with the use of Sequence Scanner version 1.0 (Copyright Applied Biosystems, Foster City, CA, USA). Nucleotide alignment was performed manually using BioEdit Sequence Alignment Editor (Hall, 1999) and using as a base the homologous sequences available on GenBank: M84604 (assemblage A), AY826193 (assemblage B), U60982 (assemblage C), U60986 (assemblage D), AY178741 (assemblage E), and AF069057 (assemblage F) for *gdh* gene and AY655704 (assemblage A, sub-assemblage AI), U57897 (assemblage A, sub-assemblage AII), AF069561 (assemblage B, sub-assemblage BIII), AF069560 (assemblage B, sub-assemblage BIV), AY228641 (assemblage C), DQ246216 (assemblage D), AY228645 (assemblage E), and AF069558 (assemblage F) for *tpi* gene.

In order to determine the phylogenetic relationship among assemblages, phylograms were constructed using Mega v.5.1 Beta, with the neighbor-joining method, with bootstrap values established in 1000 replicates.

## 3. Results

### 3.1. Positivity

Two hundred and fifty-six fecal samples from cattle, 105 from sheep, and 90 from pigs were collected. Due to the fecal collection in triplicate, 768 and 315 fecal examinations were performed in cattle and sheep, respectively. 

Positivity for *Giardia duodenalis *cysts was detected in 30 (11.7%) cattle samples, in 26 (24.8%) sheep samples, and in 3 (3.4%) pools of pig samples with a total of 59 positive samples.

### 3.2. Molecular Characterization

Out of all positive samples (*n* = 59) in three animal species, the amplification of *gdh *and *tpi* gene fragments was observed in 25 (42.4%) and 36 (61.0%) samples, respectively.

Out of 25 samples with amplified fragments of *gdh* gene, 14 were from sheep, 9 from cattle, and 2 from pigs. Out of 36 samples with amplified fragments of *tpi *gene, 15 were from sheep, 18 from cattle, and 3 from pigs.

In this study, 44 sequences were obtained and analyzed, 24 from *gdh *gene and 20 from *tpi* gene. For both genes, isolates were sequenced from the three animal species.

#### 3.2.1. *gdh*

Fourteen isolates from sheep and nine from cattle, in which *gdh *gene was sequenced, were identified as assemblage E.

When analyzing each sequence individually and comparing them to their respective base sequence, an intra-assemblage variation was seen in three isolates of sheep identified as E by the *gdh* gene. These differ from the base sequence AY178741, but they were similar among each other. Positions of nucleotide substitution were the same for the three sequences, and the nucleotides substituted at those positions were also the same (Table 1). The 11 remaining isolates of sheep were not homologous to any sequence but were identical among each other. New sequences of sheep isolates were inserted into the GenBank under the numbers KC816543 and KC816544, respectively. Regarding isolates of cattle, all of them had the same nucleotide variation (T, position 654), when compared to the reference sequence number AY178741. However, this variation made them identical to the sequence number EF07645.1 stored in the GenBank. 

**Figure 1:**
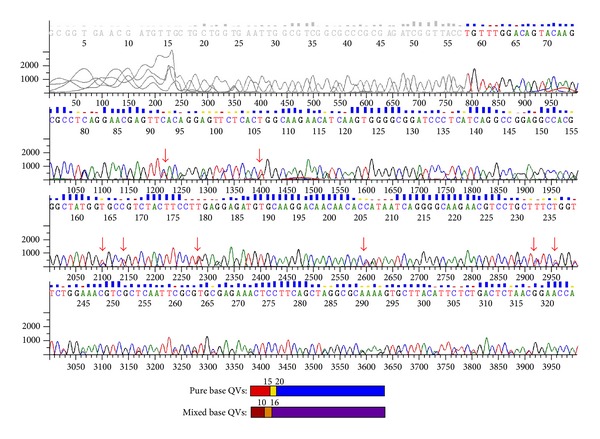
Overlapped nucleotides in chromatogram of a pig sample identified as assemblage D by the sequencing of *gdh* gene. Arrows indicate double peaks.

The two isolates of pigs differed by genotyping; one was identified as assemblage E and the other as D. The isolate identified as assemblage D was heterogeneous, with double peaks throughout the gene (Figure 1); as it was not homologous with any sequence described, it was deposited in the GenBank under the number KC816545. The isolate identified as E was identical to the reference sequence used in this study (AY178741).

**Figure 2:**
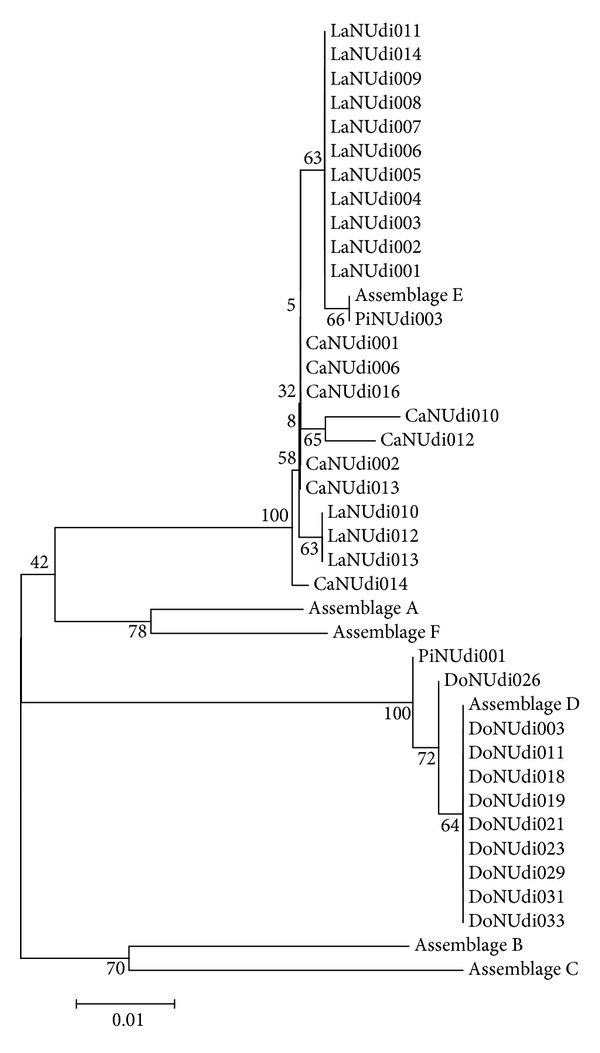
Phylogenetic relationships of *Giardia duodenalis* isolates characterized by the sequencing of *gdh *gene inferred by the neighbor-joining analysis. Bootstrap values were established in 1000 replicates.

The phylogenetic relationship among isolates genotyped by sequencing *gdh* gene is shown in the phylogram (Figure 2).

#### 3.2.2. *tpi*

Genotyping of *tpi *gene has shown variations in assemblages of 10 isolates from sheep, six identified as assemblage E, two as B, subassemblage BIII, and two as A, subassemblage AII. For cattle, eight out of nine isolates were assemblage E and one was assemblage B, subassemblage BIII. The isolate of pig was identified as a species-specific assemblage E.

Two isolates of sheep and three of cattle had polymorphic sites along the gene sequence between assemblages BIII and E, where the colocalization of conserved nucleotides was observed (Table 2).

When comparing sequences from sheep obtained in this study to those used as reference, four out of ten isolates were homologous to the reference sequence, with one being homologous to the sequence number JQ837919.1 and the others being homologous to the sequence number JQ837808.1, both stored in the Genbank. Out of the six remaining isolates of sheep, each one was different, with no homology to any sequence described, being considered new, and inserted into the Genbank under the numbers KC85814 to KC858149.

Among the sequences from cattle, one isolate was homologous to the other, present in the Genbank (JQ837925.1). The other isolates from cattle differed among each other and are present in the GenBank under the numbers KC858151 to KC858157. 

The isolate from pigs was not identical to any other described ones, thus being considered new and inserted into the Genbank under the number KC858150. 

**Figure 3:**
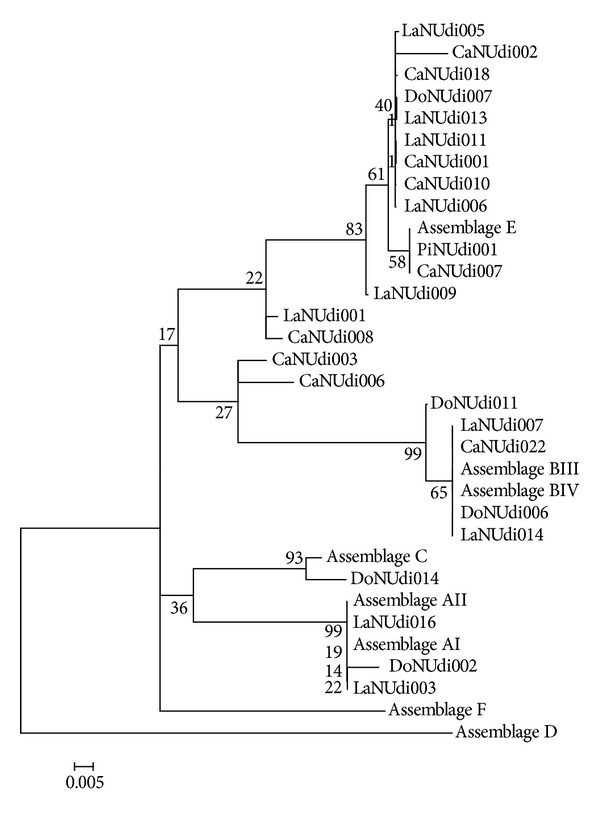
Phylogenetic relationships of *Giardia duodenalis* isolates characterized by sequencing the *tpi *gene inferred by the neighbor-joining analysis. Bootstrap values were established in 1000 replicates.

While constructing a phylogenetic tree (Figure 3), heterogeneous samples were not removed as they did not interfere with the final arrangement of the phylogram.

### 3.3. Relationship between *gdh* and *tpi* Markers When Genotyping Isolates

Out of all the samples (*n* = 61), those with amplified fragments of *gdh* and *tpi *genes, 13 (21.3%), had both genes sequenced. Among these, eight (61.5%) were in accordance with the identification of assemblage E. The other four were not in accordance with the interassemblage (Table 3). 

## 4. Discussion

The region studied is very important in the livestock production in Brazil, with 3% of the total amount of heads of cattle in the country. Nevertheless, there are no studies published on the presence and epidemiological characteristics of *Giardia duodenalis* in livestock in this region. 

PCR for both genes has failed to amplify some positive samples with the microscopic technique in this study. The failure may be attributed to the following: the presence of PCR inhibitors in the feces, the small amount of cysts, the small amount of target DNA present in the samples, and the association of these factors with the low efficiency of the DNA extraction process [16–18]. In addition, the small sample volume used and/or the loss of parasites during wash may impair the amplification [16, 19]. The choice of a target gene is also fundamentally important to the success of the amplification [20]. These genes (*gdh* and *tpi*) are less conserved and have a greater variability than other genes, which may lead to excessive imbalances in the binding sites of the nucleotide primers, resulting in a low sensitivity of the PCR [21]. 

Although authors such as Leonhard et al. [22], and Lebbad et al. [23] attribute a greater success of the amplification to the *gdh*, there is no consensus among researchers regarding which of these two genes is more effective to be the target of this reaction. In this study, *tpi* gene was more successful, being amplified in a larger number of samples when compared to *gdh*. In the present study, for three animal species, regardless of the target gene, species-specific assemblage E prevailed, which is in accordance with O'Handley et al. [9], Huetink et al. [24], Trout et al. [25], Castro-Hermida et al. [16], Souza et al. [26], Feng et al. [27]; Santín et al. [28], and Gómez-Muñoz et al. [18].

According to Read et al. [29], Wielinga and Thompson [30], and Cacciò and Ryan [21], among several genes used in the genotyping of *Giardia duodenalis* isolates, *tpi *and* gdh *provided more detailed information on *Giardia duodenalis* assemblages, as they have polymorphic sequences, which enable us to clearly distinguish genotypes. 

In the present study, it was observed that when using *gdh *gene, PiNUdi001 isolate was identified as D, which is a dog-specific assemblage. With the chromatogram of this sample, in strategic sites along the gene, double peaks were seen, which characterizes a possible mixed infection. Overlapped nucleotides are located on the same positions of assemblages D and E, which suggests the concomitant presence of both assemblages in the isolate. Although rare, these findings were also observed by Langkjær et al. [31] and Sprong et al. [32].

The sequencing of the *gdh *gene has also shown differences between the reference sequence (assemblage E) and three isolates from sheep, identified as E. The pattern of nucleotide substitution, associated with the analysis of the phylogenetic tree, suggests that these isolates are subassemblages of assemblage E. The position of isolates on the tree is the basis of this hypothesis, as they are grouped in a branch of the tree that is different from the one which gathers other isolates from the same assemblage. Some studies postulate the existence of subassemblages E [33, 34], although there are no factual data supporting this. According to Yang et al. [35], when the result is obtained by a single gene locus, data are not conclusive, as this pattern was observed only when genotyping *gdh*. Thus, further studies are needed for confirmation.

When genotyping *tpi*, this gene was seen to have a greater variability of interassemblage when compared to *gdh*. This result is in agreement with findings of Wielinga and Thompson [30], Cacciò and Ryan [21], and Gómez-Muñoz et al. [18]. In this study, in addition to assemblage E, *tpi* has also identified zoonotic assemblages BIII in cattle and sheep and AII in sheep. Sulaiman et al. [7] identified *tpi* gene as the most informative to genotype isolates of *Giardia duodenalis*. This characteristic becomes evident over the entire gene, both regarding interspecies (*Giardia* spp.) and intraspecies (*Giardia duodenalis*), which may be seen in the number of new sequences identified. In this study, *tpi* has shown 15 new sequences and just two for *gdh*, reinforcing the comments made by Wielinga and Thompson [30], Cacciò and Ryan [21], and Gómez-Muñoz et al. [18] who highlighted that *tpi* is an important target of research in order to identify isolates and to study outbreaks [18]. 

Heterogeneous sequences have been observed when genotyping *tpi* and a larger number of double peaks were observed over the entire gene when compared to *gdh*, which is in accordance with the results obtained by Lebbad et al. [23]. Overlapped nucleotides were observed in isolates identified as BIII and E, which suggest a possible mixed infection, BIII/E and E/BIII. Nevertheless, the presence of double peaks in the chromatogram may not always be described as a mixed infection; thus, hypotheses such as the one of meiotic recombination and that of allelic heterozygosity [21] should also be considered. For more consistent results, Levecke et al. [36], Lebbad et al. [23], and Almeida et al. [37] suggest the use of assemblage-specific primers, which are able to distinguish more precisely intra-assemblage infections. Double peaks have been frequently reported in isolates of assemblages B, C, D, and E but never in isolates of assemblages A, F, and G. Cacciò and Ryan [21] and Lalle et al. [38] reported that double peaks are more common in subassemblages B, due to the high level of allelic divergence of the sequence. In the present study, more isolates of assemblage E have been observed with double peaks, which is in agreement with the findings of Lebbad et al. [23]. There are no reports in the literature regarding the simultaneous presence of assemblages B and E in the same isolate, but Yang et al. [35], Feng et al. [27], and Santín et al. [28] reported the existence of mixed infections between assemblages A and E in livestock. Gelanew et al. [17] mentioned the existence of mechanisms such as introgression, which is likely to bring about changes of assemblages when different genes are used. Smith et al. [39] suggest that the occurrence of mixed infections in several assemblages and subassemblages of *G. duodenalis *reflects the complex behavior of the parasite in the environment and the exposure of humans and animals to multiple sources.

Studies have reported that 15% of isolates genotyped have genotypic inconsistencies between two markers [7]. In the present study, the inconsistency rate was 38.5%. Samples with a complete interassemblage concordance for both genes were identified as E, confirming the predominance of species-specific assemblage in livestock. In most conflicting cases, the presence of subassemblages AII and BIII was seen at the same proportions. According to Read et al. [29] and Leonhard et al. [22], this phenomenon has been found in human and animal isolates, and it seems to be frequent in the combination of different marker genes, when an isolate may be genotyped as zoonotic by a primer and species-specific by another. For molecular epidemiological studies, there are major implications on the inferences from the conclusions depending on the interpretations of the data obtained.

Genetics of the *Giardia duodenalis* complex has not yet been fully elucidated. Understanding the microepidemiology of giardiasis requires characterizing the sources of contamination and understanding the role of anthroponotic, zoonotic, and environmental transmissions. The choice of the primer may directly influence the results; thus, assemblage-specific primers should be included, mainly when analyzing mixed infections and data should be carefully interpreted.

## Figures and Tables

**Table 1:** Variation of *Giardia duodenalis *intra-assemblage E from three isolates of sheep from the microregion of Uberlândia identified by the *gdh* gene.

Sample	Positions			
	520	585	630	654
AY178741*	C	G	C	A
LaNUdi010	T	A	T	G
LaNUdi012	T	A	T	G
LaNUdi013	·	A	T	G

*Base sequence of assemblage E stored at GenBank.

**Table 2:** Overlapped nucleotides in isolates from cattle and sheep characterized as E and BIII genotyping by *tpi*.

	Isolates						
	AF069561*	AY228645*	LaNUdi001**	LaNUdi007***	CaNUdi003**	CaNUdi006**	CaNUdi008**
Nucleotides positions and replacements							
347	C	T	T	·	·	·	·
350	C	T	A	·	·	·	·
374	G	A	A	·	·	·	·
396	C	T	T	T	·	·	·
397	T	C	C	·	·	·	·
398	T	C	C	·	·	·	·
405	C	T	·	T	·	·	·
408	T	A	A	·	·	·	·
411	T	A	A	·	·	·	·
426	G	A	A	A	·	·	·
429	T	C	C	C	·	·	·
432	T	G	G	·	·	·	·
439	T	T	C	·	·	·	·
440	G	A	A	·	·	·	·
442	C	T	T	·	·	·	·
462	C	T	T	·	·	·	·
468	T	C	C	·	·	·	·
471	G	A	A	·	·	·	·
473	A	G	G	·	·	·	·
483	T	A	A	·	·	·	·
495	C	T	T	·	·	·	·
501	A	G	·	·	A	A	A
504	C	T	T	·	·	·	·
507	C	T	T	·	·	C	·
518	T	A	A	·	·	·	·
528	G	C	·	·	T	T	·
529	C	T	T	·	·	·	·
532	A	A	A	·	A	A	·
533	G	A	·	·	·	G	·
534	C	G	·	·	C	C	·
535	C	T	·	·	·	C	C
537	T	C	·	·	T	T	T
540	A	G	·	·	A	A	·
548	G	G	·	·	A	A	·
549	A	G	G	·	·	·	·
552	C	T	T	·	·	C	·
561	A	G	·	·	A	A	·
583	A	G	·	·	A	·	·
585	T	C	·	·	T	·	·
597	T	C	C	·	·	·	·
606	G	T	T	·	·	·	·
618	G	A	·	·	G	G	G
624	A	G	·	·	·	A	·
625	G	G	·	·	·	·	A
627	T	G	·	·	T	T	T
633	T	G	G	·	·	·	·
642	C	T	·	·	·	C	·
645	C	T	·	·	C	C	·
647	C	T	G	·	·	·	·
693	A	A	·	G	·	·	·

*AF069561: assemblage BIII; *AY228645: assemblage E; **isolates identified as assemblage BIII, ***isolates identified as assemblage E; dots indicate identity to the reference sequences.

**Table 3:** Genotyping and subgenotyping of samples from sheep, pigs, and cattle from the sequencing of *gdh *and* tpi *genes.

Samples	* gdh *	*tpi *	
	Assemblage	Assemblage	Subassemblage
LaNUdi001	E	E	
LaNUdi002	E	—	
LaNUdi003	E	A	II
LaNUdi004	E	—	
LaNUdi005	E	E	
LaNUdi006	E	E	
LaNUdi007	E	B	III
LaNUdi008	E	—	
LaNUdi009	E	—	
LaNUdi010	E	E	
LaNUdi011	E	B	III
LaNUdi012	E	—	
LaNUdi013	E	—	
LaNUdi014	E	A	II
LaNUdi015	—	E	
LaNUdi016	—	E	
PiNUdi002	E	—	
CaNUdi001	E	E	
CaNUdi002	E	E	
CaNUdi003	—	E	
CaNUdi005	—	E	
CaNUdi006	E	E	
CaNUdi008	—	E	
CaNUdi010	E	E	
CaNUdi012	E	—	
CaNUdi013	E	—	
CaNUdi014	E	—	
CaNUdi016	E	—	
CaNUdi018	—	E	
CaNUdi022	—	B	III

La: lambs, Ca: cattle, Pi: pig.
